# Assessment of computational methods for fusion transcript detection in targeted RNA-seq samples

**DOI:** 10.3389/fbinf.2026.1695973

**Published:** 2026-10-28

**Authors:** Lucas P. Oliveira, Victor Rigatto, Gustavo M. C. Alves, Natacha A. Migita, José A. Yunes, João Meidanis

**Affiliations:** 1 Institute of Computing, University of Campinas, Campinas, Brazil; 2 Institute of Biology, University of Campinas, Campinas, Brazil; 3 Boldrini Children’s Center, Campinas, Brazil; 4 Department of Translational Medicine, University of Campinas, Campinas, Brazil

**Keywords:** benchmarking, cancer, gene fusion, RNA-seq, transcript fusion

## Abstract

Transcript fusions are abnormal genetic events often correlated with oncogenesis. Hence, detecting them from RNA-seq data using bioinformatics methods is an important task in cancer research. Several tools have been developed for this task, but current benchmarks are inconclusive regarding their accuracy in real data. Moreover, they have used only bulk RNA-seq samples on their assessments, and current transcript fusion detection methods have not yet been assessed under alternative settings, such as on targeted RNA-seq samples. In this article, we assess 8 transcript fusion detection tools (Arriba, ChimeRScope, CICERO, FuSeq, FusionCatcher, Pizzly, JAFFA, and STAR-Fusion) on targeted RNA-seq samples and simulated bulk RNA-seq samples. We selected the best evaluated tools in previous benchmarks. We also divided them into alignment-based and alignment-free. The targeted RNA-seq dataset comprises 92 samples of real pediatric B-cell precursor acute lymphoblastic leukemia cases with manually validated fusions. The simulated dataset comprises 10 samples with synthetic fusions. Our results point out that STAR-Fusion and FusionCatcher had superior performance on the targeted RNA-seq samples, while Arriba and STAR-Fusion had superior performance on the simulated samples. Overall, we conclude that STAR-Fusion is the most broadly robust tool across both settings.

## 1 Introduction

Gene fusions arise from chromosomal rearrangements that join distinct genes into a hybrid gene, which may give rise to chimeric transcripts and, in some cases, aberrant proteins with oncogenic potential. This genetic event is an important biomarker to diagnose and treat various types of cancer. RNA sequencing (RNA-seq) has proved to be the most cost-effective technology to detect gene fusions because it is more accessible than whole-genome sequencing and one focuses on the gene fusions with functional effect. At the RNA level, these gene fusions can yield fused transcripts, i.e., chimeric transcripts consisting of pieces of the transcripts of the original genes, and this is the biomarker that is sought after by current bioinformatics methods.

Several tools have been developed to detect transcript fusions from short-read RNA-seq data, and multiple studies have assessed them on simulated and real data (Carrara et al., 2013; Liu et al., 2016; Kumar et al., 2016; Singh and Li, 2021; Creason et al., 2021; Haas et al., 2019). The results often agree on simulated data, but are inconclusive on real data because no truth set can be perfectly defined. Moreover, a tool’s performance may vary depending on the transcript fusions contained in the sample. For instance, transcript fusions that originate from gene fusions in complex regions, e.g., IGH and DUX4 loci, are often missed by current tools. Finally, current studies use four breast cancer cell lines—BT474, KPL4, MCF7, and SKBR3 — as standard benchmark datasets, but they are not representative for benchmarking purposes. These cell lines have been in continuous culture for decades and are known to accumulate substantial chromosomal instability and inter-clonal heterogeneity beyond what was present in the original tumors (Rondón-Lagos et al., 2014), raising the possibility that some benchmarked fusions reflect culture-acquired artifacts rather than clinically relevant events. Moreover, these 4 cell lines represent a single cancer type and yield a comparatively small validated truth set, limiting their ability to capture the diversity of fusion partners, breakpoint structures, and expression contexts seen across cancers. They also derive from bulk RNA-seq, which differs substantially from the targeted RNA-seq setting this article addresses (Maher et al., 2009; Edgren et al., 2011; Asmann et al., 2011; Kangaspeska et al., 2012).

In this article, we assess 8 transcript fusion detection tools—Arriba, STAR-Fusion, FusionCatcher, FuSeq, Pizzly, ChimeRScope, JAFFA, and CICERO (Uhrig et al., 2021; Haas et al., 2019; Nicorici et al., 2014; Vu et al., 2018; Melsted et al., 2017; Li et al., 2017; Davidson et al., 2015; Tian et al., 2020) — on the 2 simulated bulk RNA-seq datasets of Haas et al. (2019), each comprising 5 samples, and a real targeted RNA-seq dataset comprising 92 samples of B-cell precursor acute lymphoblastic leukemia (BCP-ALL) cases (Migita et al., 2023). Instead of bulk RNA-seq samples (such as the aforementioned breast cancer cell lines), the cancer samples we assess here comprise RNA-seq samples of selected regions spanning 1,385 cancer-related genes. These samples are separated into two subtypes depending on the transcript fusions they harbor: the classical ones comprise well-characterized transcript fusions, while the so-called B-other ones comprise a broader diversity of transcript fusions.

To the best of our knowledge, no benchmark has assessed the performance of these tools on targeted RNA-seq datasets. Since this technology is more accessible for a clinical setting, the assessment we provide in this article may be of interest to practitioners in this context. We selected the best evaluated tools in previous benchmarks and added CICERO, a tool that is very popular among practitioners but has not been assessed in previous benchmarks. We also considered both alignment-based and alignment-free tools, to offer a wide view of the landscape. To compare these tools, we developed a pipeline that leverages utilities from the Fusion Transcript Benchmark Toolkit (Haas et al., 2019); optionally, it can also be used as an ensemble approach for fusion detection, although simplistic, as it does not implement a consensus algorithm.

This article is the extended version of the one published in the Proceedings of the XVII Brazilian Symposium on Bioinformatics (Oliveira et al., 2024). In the previous version, we analyzed 48 samples of the targeted RNA-seq datasets, whereas here we included all the samples from the study of Migita et al. (2023) that harbored fused transcripts. In addition, we included JAFFA in this extended version, expanded the analysis of the results, and revised the overall presentation. The article is organized as follows: We describe the transcript fusion detection tools in Section 2.1; the simulated datasets in Section 2.2; the targeted RNA-seq datasets in Section 2.3; the pipeline in Section 2.4; the performance metrics in Section 2.5; the results on simulated and real datasets in Sections 3.1 and 3.2, respectively; and, finally, we discuss these results in Section 4 and summarize them in Section 5.

## 2 Materials and methods

### 2.1 Fusion detection software packages

Table 1 summarizes the fusion detection methods used in this study. We selected five alignment-based methods—Arriba, STAR-Fusion, FusionCatcher, JAFFA, and CICERO—and three alignment-free methods—FuSeq, Pizzly, and ChimeRScope (Uhrig et al., 2021; Haas et al., 2019; Nicorici et al., 2014; Davidson et al., 2015; Tian et al., 2020; Vu et al., 2018; Melsted et al., 2017; Li et al., 2017). These methods seek two signatures that may indicate a fusion event: junction reads and spanning reads. The former occurs when a single read spans the breakpoint and aligns partially to each fusion partner; the latter, in the case of paired-end reads, occurs when each read of the pair maps to different genes, together covering the breakpoint region.

**TABLE 1:** Fusion detection methods for short-read RNA-seq data compared in this study, alongside with the version used and other characteristics.

Method (version)	Article	Reference	Read mapper
Arriba (v2.4.0)	Uhrig et al. (2021)	Genome	STAR (raw reads)
CICERO (v1.9.5)	Tian et al. (2020)	Genome	STAR (raw reads) and BLAT (assembled contigs)
STAR-fusion (v1.10.1)	Haas et al. (2019)	Genome	STAR (raw reads)
FuSeq (v1.1.4)	Vu et al. (2018)	Transcriptome	RapMap*
Pizzly (v0.37.3)	Melsted et al. (2017)	Transcriptome	–
ChimeRScope (git 6eee2a8)	Li et al. (2017)	Transcriptome	–
JAFFA (v2.5)	Davidson et al. (2015)	Transcriptome	Bowtie2 (raw reads) and BLAT (assembled contigs)
FusionCatcher (v1.33)	Nicorici et al. (2014)	Transcriptome and genome	Bowtie2 (raw reads against transcriptome) and STAR, bowtie, Bowtie2, and BLAT (second mapping against genome)

In all cases, the reference was the Human genome version 38 revision 111.

* RapMap can be considered a read mapper, but it does not perform base-to-base alignment like the other read mappers in this table.

The alignment-free methods index the k -mers of a reference transcriptome and associate them with their originating transcripts. Raw reads are then classified as fusion supporting or not based on their k -mer composition after searching them against the index. In contrast to traditional base-to-base alignment, this search attempts to identify the transcripts from which the reads have originated without pinpointing how exactly the sequences of the reads and transcripts align. This is the approach underlying FuSeq, Pizzly, and ChimeRScope, although its implementation differs significantly among these methods. Pizzly leverages the pseudoalignment procedure from kallisto to map reads and identify spanning reads that may indicate a fusion event (Melsted et al., 2017; Bray et al., 2016). FuSeq employs a similar approach, but instead of pseudoalignment, it uses the quasi-mapping procedure from RapMap to map reads and then flag junction reads (Vu et al., 2018; Srivastava et al., 2016). ChimeRScope builds a graph network model on top of the k -mer content of a read and assigns to it a score that is maximized when the read contains enough k -mers from distinct transcripts (Li et al., 2017).

Alignment-based methods map the raw reads against a reference genome or transcriptome through base-to-base alignment to identify chimeric alignments that may indicate a fusion event. In this category, Arriba and STAR-Fusion have been consolidated as the top-performing methods in most simulated short-read RNA-seq datasets (Haas et al., 2019; Creason et al., 2021; Singh and Li, 2021). Both optimize the parameters of the STAR aligner and map the reads against a reference genome to retrieve the chimeric alignments of junction and spanning reads (Dobin et al., 2013). These alignments then undergo extensive filtering steps to remove artifacts and false positives (Uhrig et al., 2021; Haas et al., 2019). CICERO employs a similar approach, but, in addition to the conventional alignment step, it also locally assembles a subset of soft-clipped reads using CAP3 and maps them against the reference genome using BLAT (Huang and Madan, 1999; Kent, 2002; Tian et al., 2020). This local assembly step allows the detection of non-canonical fusion events, e.g., tandem duplications and rearrangements in highly repetitive regions such as the immunoglobulin loci.

JAFFA and FusionCatcher, on the other hand, map the reads against a reference transcriptome and, in the case of FusionCatcher, also against a reference genome for filtering purposes (Nicorici et al., 2014; Davidson et al., 2015). JAFFA is a multi-step pipeline that provides multiple running modes for the mapping step that the user adjusts depending on the read length. For reads shorter than 99 bp, ‘Assembly’ mode assembles them into transcripts before the mapping step; for reads 60 to 99 bp long, ‘Hybrid’ mode assembles them and also adds to the list of contigs the reads that do not map to either the reference or the assembly; and for reads 100 bp long and over, ‘Direct’ mode maps them directly against the reference (Davidson et al., 2015). We used the appropriate mode for each of our datasets depending on their read lengths. When mapping raw reads and assembled transcripts against the reference transcriptome, JAFFA uses the Bowtie2 and BLAT aligners, respectively (Langmead and Salzberg, 2012; Kent, 2002). Finally, FusionCatcher maps the raw reads against a reference transcriptome using the Bowtie2 aligner and identifies fusion junctions by mapping the unmapped reads against annotation databases. Specifically, it maps the unmapped reads against a database of exon-exon junctions using the Bowtie aligner; then, reads still unmapped after this step are further mapped against a database of gene-gene sequences using the STAR, Bowtie2, and BLAT aligners (Kent, 2002; Langmead et al., 2009; Langmead and Salzberg, 2012; Dobin et al., 2013).

### 2.2 Simulated dataset

We used the simulated datasets of Haas et al. (2019), as generated by their Fusion Transcript Benchmark Toolkit (Haas et al., 2019). There are two datasets comprising a total of ten samples that simulate 30 million paired-end (PE) Illumina RNA-seq reads; each sample has 500 fused transcripts previously annotated. One dataset comprises five simulated samples of 50 bp reads (PE50) and the other comprises five simulated samples of 101 bp reads (PE101). The former was based on short RNA-seq reads generated by the Illumina Human Body Map 2.0 study, specifically the ArrayExpress study E-MTAB-513 (Schroth, 2011); the latter was based on a set of cancer cell lines from the Cancer Cell Line Encyclopedia (CCLE) (Barretina et al., 2012).

Unlike the targeted RNA-seq dataset (Section 2.5), reciprocal fusion orientations (A::B and B::A) were treated as equivalent for scoring purposes in the simulated datasets, to remain consistent with the methodology of prior benchmarks using this data (Haas et al., 2019).

### 2.3 Targeted RNA-seq dataset

We assessed fusion prediction accuracy using 92 samples that comprise a cohort of B-cell precursor acute lymphoblastic leukemia (BCP-ALL) cases (Migita et al., 2023). We selected the samples harboring fused transcripts and excluded the ones of high hyperdiploidy or hypodiploidy ALL cases, which usually correspond to 30% of ALL cases (Migita et al., 2023). These samples were generated from targeted RNA-seq of 1,385 cancer-related genes using the TruSight RNA Pan-Cancer kit (Illumina); each sample has approximately 3 million paired-end 76 bp reads.

The truth set was constructed from Supplementary Table S4 of Migita et al. (2023), which lists candidate fusion transcripts per sample together with their validation status. We excluded entries where either gene partner was an unidentified locus (NA), an open reading frame (ORF) designation, or a LOC-prefixed identifier, as these do not correspond to named genes suitable for benchmarking. We further excluded fusions involving the JSRP1/OAZ1/DOT1L locus, where JSRP1 and OAZ1 are consecutive, overlapping genes on opposite strands not resolvable by short-read fusion detection, and fusions involving IGH, whose highly repetitive, somatically rearranging sequence is intractable for the tools benchmarked here. Applying these criteria yields 62 distinct fusion transcripts across the 92 samples, of which 29 were independently confirmed by an orthogonal method (FISH, RT-PCR, or MLPA); the remaining 33 were detected by at least three computational methods but were not selected for orthogonal validation, as they were considered clinically irrelevant in the original study. The complete sample list, their transcript fusions, and validation methods are available as Supplementary Material.

The samples were categorized as either classical, harboring long-established transcript fusions, or B-other, harboring different transcript fusions or none. Within each of these categories, the samples were further divided into the following groups according to the type of transcript fusions therein:

- Classical (37): BCR-ABL1 (4), ETV6-RUNX1 (24), TCF3-PBX1 (3), and KMT2A-rearrangements (6);
- B-other (55): ABL-class (7), JAK2-rearrangements (3), P2RY8-CRLF2 (6), ZNF384-rearrangements (9), NUTM1-rearrangements (4), PAX5-rearrangements (9), B-rest (5), DUX4-rearrangements (7), MEF2D-rearrangements (4), and iAMP21 (1).

This classification is based on Supplementary Table S5 of Migita et al. (2023), with the following exceptions: sample BCPALL-1518, which has both JAK2:PAX5 and PAX5:JAK2 fusion, was classified in the JAK2-r family, following what was done in the original study; and BCPALL-0597 and BCPALL-2178, which do not appear in Supplementary Table S5 of Migita et al. (2023), were classified as B-rest.

### 2.4 Computational pipeline

We developed a pipeline that executes the tools and parses their outputs into a standard format. It then adds extra annotations to the predicted fusions and scores them using the scheme presented in Section 2.5; these steps leverage some utilities from the Fusion Transcript Benchmark Toolkit (Haas et al., 2019), which we explain in the sequel. We executed the pipeline in a machine running Ubuntu version 22.04 with two 2.10 GHz Intel(R) Xeon(R) Gold 6530 CPU (totaling 128 cores) and 512 GB of RAM. Figure 1 depicts a diagram of the pipeline in which each box represents a step numbered by their order of execution. In this section, we elaborate on each of the blue tasks by referring to their numberings.

1. The tools shown in Table 1 were executed with default parameters. Their execution times in the PE50 and PE101 datasets were measured using the/usr/bin/time utility, once per sample; the results of this experiment are available in the Supplementary Material. For the alignment-based methods, we remark that some of them expect different parameters from the STAR aligner; hence, we set the parameters as described in the tools’ documentations. The hg38 reference—genome and transcriptome—was used for all tools.
2. After the fusion calling step, the predicted fusions and their number of supporting reads (SR) were gathered from the tools’ outputs. All tools report the number of junction reads and spanning reads that support the predictions, except Pizzly and FuSeq, which count just one of these quantities. When both are available, the number of SRs is their sum; otherwise, it is one of the two depending on the tool at hand. The only exception is ChimeRScope, which uses a different notion of supporting read (see Section 2.1).
3. This step leverages utilities from the Fusion Transcript Benchmark Toolkit (Haas et al., 2019). After parsing the tools’ outputs, the gene symbols of the predicted fusions were mapped to the GENCODE v19 annotation set (Frankish et al., 2022). This is justified by two reasons. First, each tool may use a different annotation resource to report the gene symbols of a predicted fusion and they may not match those of the GENCODE v19 annotation set. Second, considering that genes may overlap on the same genomic coordinate, different tools may report different transcripts for the same transcript fusion based on similar (if not identifical) fusion events. To account for these inconsistencies, the mapping assigns to each transcript of a predicted fusion alternative gene symbols from the GENCODE v19 annotation set as well as gene symbols of other genes that overlap with their genomic coordinates in the hg19 coordinate system. For example, if the predicted fusion is A–B, A has an alternative gene symbol $A^{\prime }$ , and a gene C overlaps A’s coordinate in hg19, then the fusions $A^{\prime }$ –B and C–B are considered valid proxies for A–B.
4. FusionAnnotator (Haas et al., 2023) adds metadata to the annotation of the predicted fusions, e.g., whether the fusion is intra- or inter-chromosomal, the origin of the genes, the databases where the fusion has been recorded, etc.
5. The metadata added in the preceding step are used to filter transcript fusions of ambiguous origin: those involving neighboring genes, sequence-similar genes, mitochondrial genes, and HLA genes. Self-fusions (i.e., fusions of the form X–X) and transcript fusions with less than 10 supporting reads were also filtered out. We observed that some tools report far more fusions with ≤10 SRs than informative fusions, which we considered safe to discard in our analyses.
6. See Section 2.5.
7. The metrics described in Section 2.5 were summarized in the graphs that we discuss in Section 3.

**FIGURE 1:**
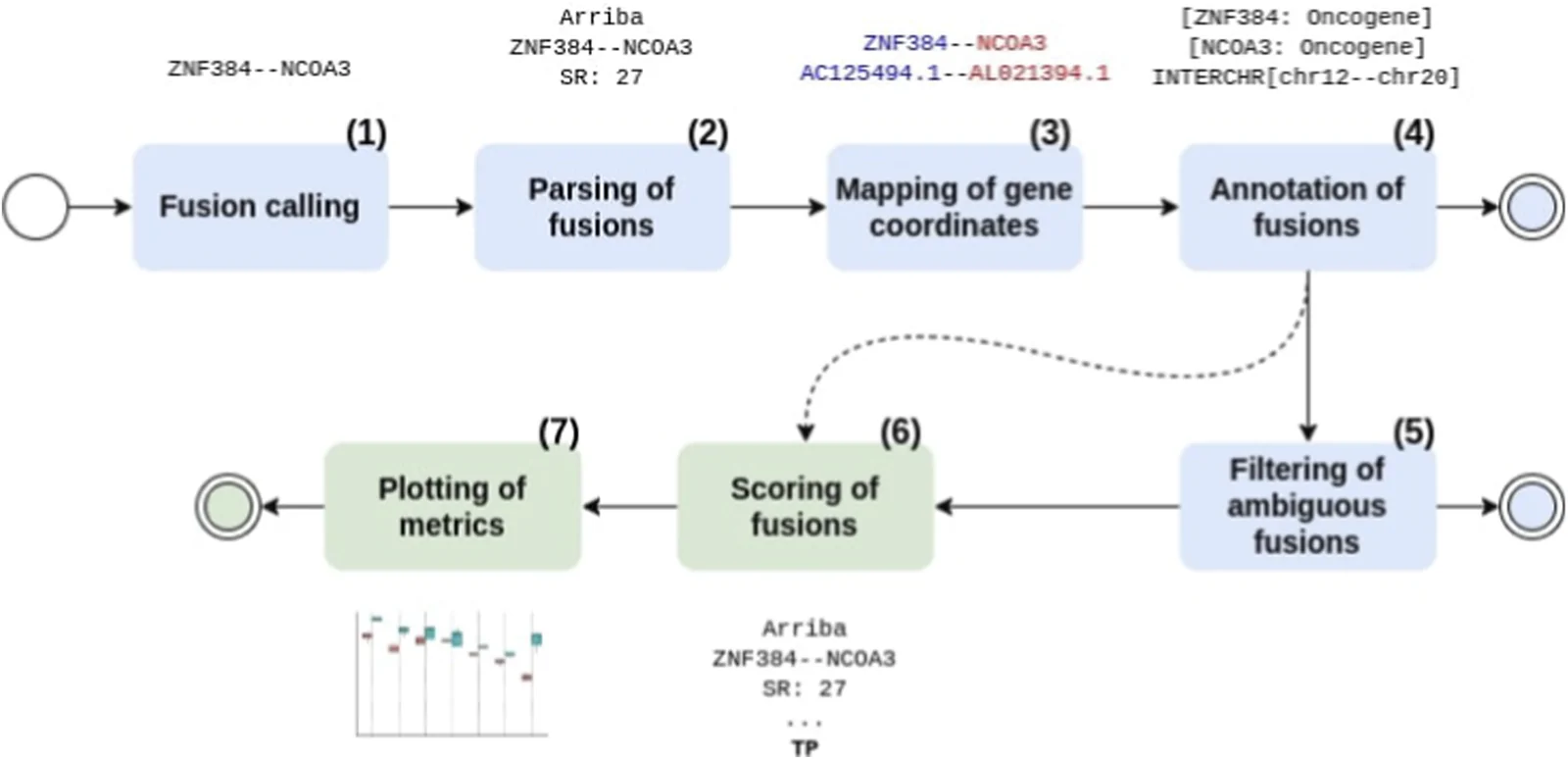
Computational pipeline to detect fusions and benchmark tools. Each box represents a step, the white circle represents the starting point, and filled circles represent ending points. The blue steps, from (1) to (4), constitute an ensemble transcript fusion detection pipeline, which may include step (5) for filtering. Green steps perform the benchmark by scoring the tools and plotting the results. For the sake of example, the steps are labeled with a simplified view of their outputs for the fusion *ZNF384::NCOA3* retrieved by Arriba.

We remark that the pipeline, up to step (5), can be used as an ensemble fusion detection method, although naive because no consensus algorithm is applied.

### 2.5 Performance metrics

True positive (TP), false positive (FP), and false negative (FN) fusion predictions were assessed for each tool using the following scoring scheme, available in the Fusion Transcript Benchmark Toolkit (Haas et al., 2019). Consider genes A and B with paralogs $A^{\prime }$ and $B^{\prime }$ , and mapped genes $A^{\prime \prime }$ and $B^{\prime \prime }$ under the GENCODE v19 annotation set (as described in Section 2.4), respectively. Then, a predicted transcript fusion A–B was scored as follows:

- TP: the truth set includes either A–B, $A^{\prime }$ – $B^{\prime }$ , $A^{\prime \prime }$ – $B^{\prime \prime }$ , A– $B^{\prime }$ , A– $B^{\prime \prime }$ , etc.,;
- FP: the truth set does not include any of the combinations described above.

On the other hand, if A–B is a TP according to the preceding definition but was not predicted by the tool being evaluated, then it was scored as FN. Moreover, true positive rate (TPR; a. k.a. recall or sensitivity), positive predictive value (PPV; a. k.a. precision), and F1-score (the harmonic mean of TPR and PPV) were measured as follows:

$$\begin{array}{ll} TPR & =TP/\left(TP+FN\right),\\ PPV & =TP/\left(TP+FP\right),\\ F1 & =2\times \left(TPR\times PPV\right)/\left(TPR+PPV\right). \end{array}$$

The area under the curve (AUC) of the precision-recall (PR) curve was also computed; the threshold used was the minimum number of supporting reads to call the fusions. For example, if a tool predicts four transcript fusions whose number of supporting reads are 100, 75, 50, and 25, then the PR curve has four points for PPV and TPR measurements at those thresholds: at 100, they are measured for one predicted fusion; at 75, they are measured for two predicted fusions; and so on. As this threshold decreases, a tool with good accuracy should report more fusions without decreasing the AUC, i.e., PPV and TPR should remain stable. The AUC of the PR curves for each tools was computed on each dataset over 100 iterations, each one sampling 50% of the samples in the dataset uniformly at random. We also repeated this experiment for different sampling rates — 25%, 50%, 75%, and 100% — and number of iterations — 50, 100, and 200 —, but for every combination, the relative performance of the tools did not change.

Pairwise differences in mean AUC between tools were assessed using two-sided Mann-Whitney U tests on the 100 bootstrap iteration values per tool, with Benjamini–Hochberg correction for multiple comparisons across all pairwise tests within each dataset.

For fusions detected as true positives by two or more tools, we additionally assessed agreement at the level of predicted genomic breakpoint coordinates. Six of the eight tools report a specific genomic breakpoint for each predicted fusion (Arriba, STAR-Fusion, CICERO, FusionCatcher, FuSeq, and JAFFA); Pizzly and ChimeRScope, both alignment-free, report no genomic coordinate and were excluded from this analysis. Where a tool reported multiple candidate breakpoints for the same gene pair, the one with the highest number of supporting reads was used, matching the criterion by which the scoring pipeline itself selects a tool’s true positive call. For each multi-tool-detected fusion, breakpoints were clustered using a 5bp tolerance, and a tool was considered an outlier if its reported position fell outside the largest resulting cluster.

## 3 Results

The mean AUC of PR curves of the tools is shown in Figure 2 for the targeted RNA-seq, PE50, and PE101 datasets. Moreover, Figure 3, Figures 4, 5 show the TPR, PPV, and F1-score for the tools across the targeted RNA-seq, PE50, and PE101 datasets, respectively. For the targeted RNA-seq dataset, we do not distinguish the samples by category (Classical or B-other, and the groups therein) in Figure 3. This distinction is made in Figures 6, 7, which show FP, TP and FN counts per sample, colored by the groups described in Section 2.3. For TP and FN counts, we also show the validated transcript fusions in each sample. In Sections 3.1 and 3.2, we elaborate on the results for the simulated and real datasets, respectively.

**FIGURE 2:**
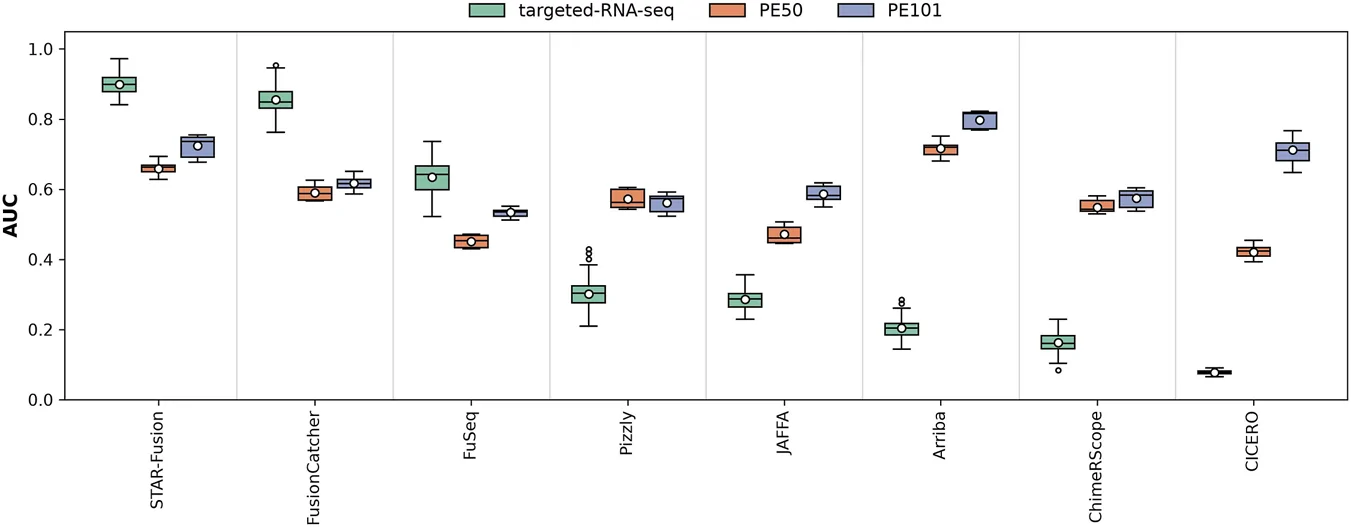
Mean area under the curve (AUC) of precision-recall (PR) curves for each tool in targeted RNA-seq, PE50, and PE101 datasets over 100 random samplings, each selecting 50% of the samples from each dataset. The threshold used for computing the metrics was the minimum number of supporting reads to call the fusions.

**FIGURE 3:**
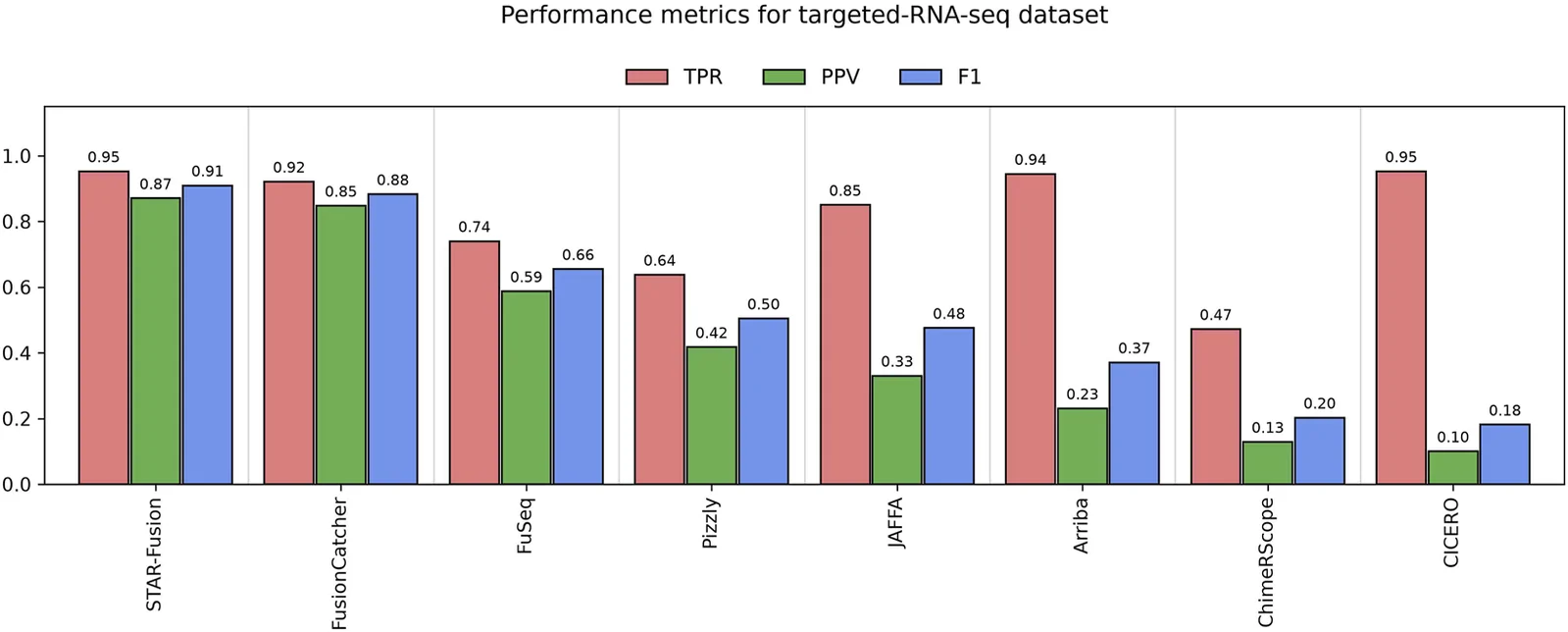
TPR, PPV, and F1-score for the targeted RNA-seq dataset (encompassing the Classical and B-other datasets), which contains 62 transcript fusions across 92 samples.

**FIGURE 4:**
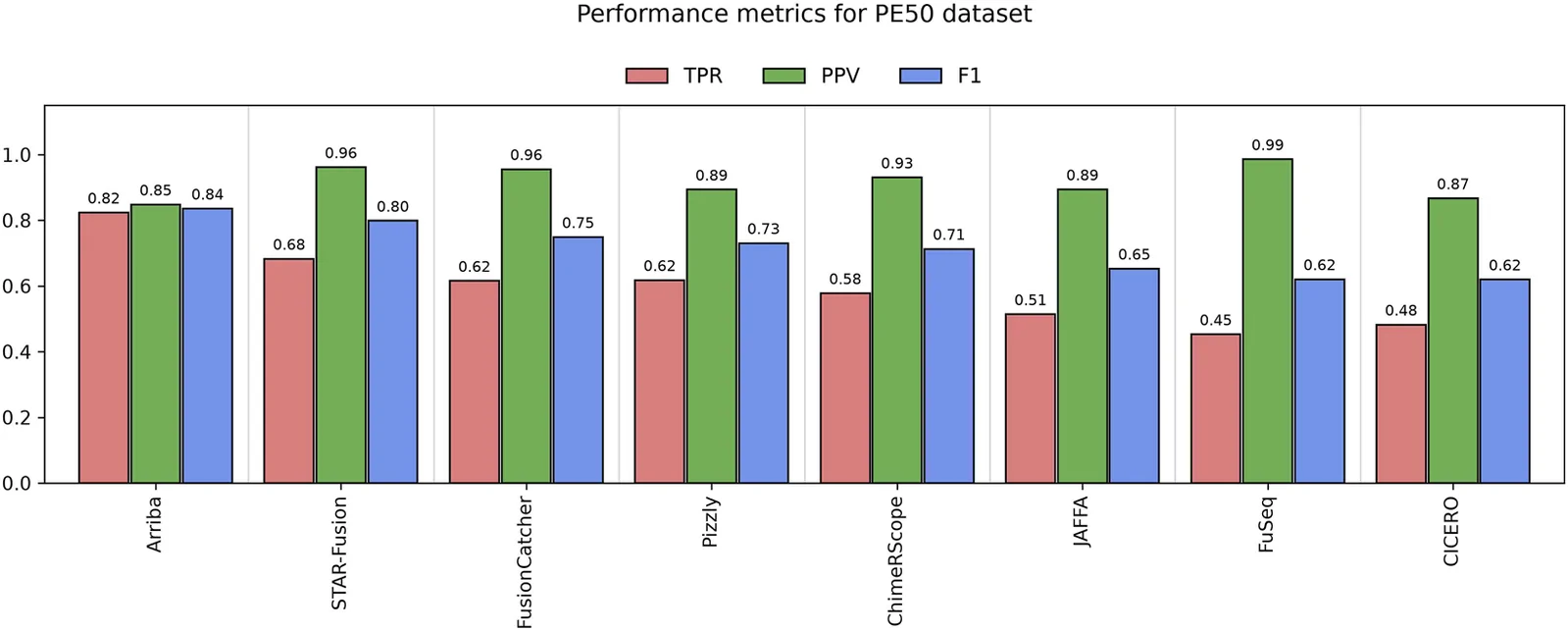
TPR, PPV, and F1-score for the simulated PE50 dataset, which contains 500 transcript fusions across 5 samples.

**FIGURE 5:**
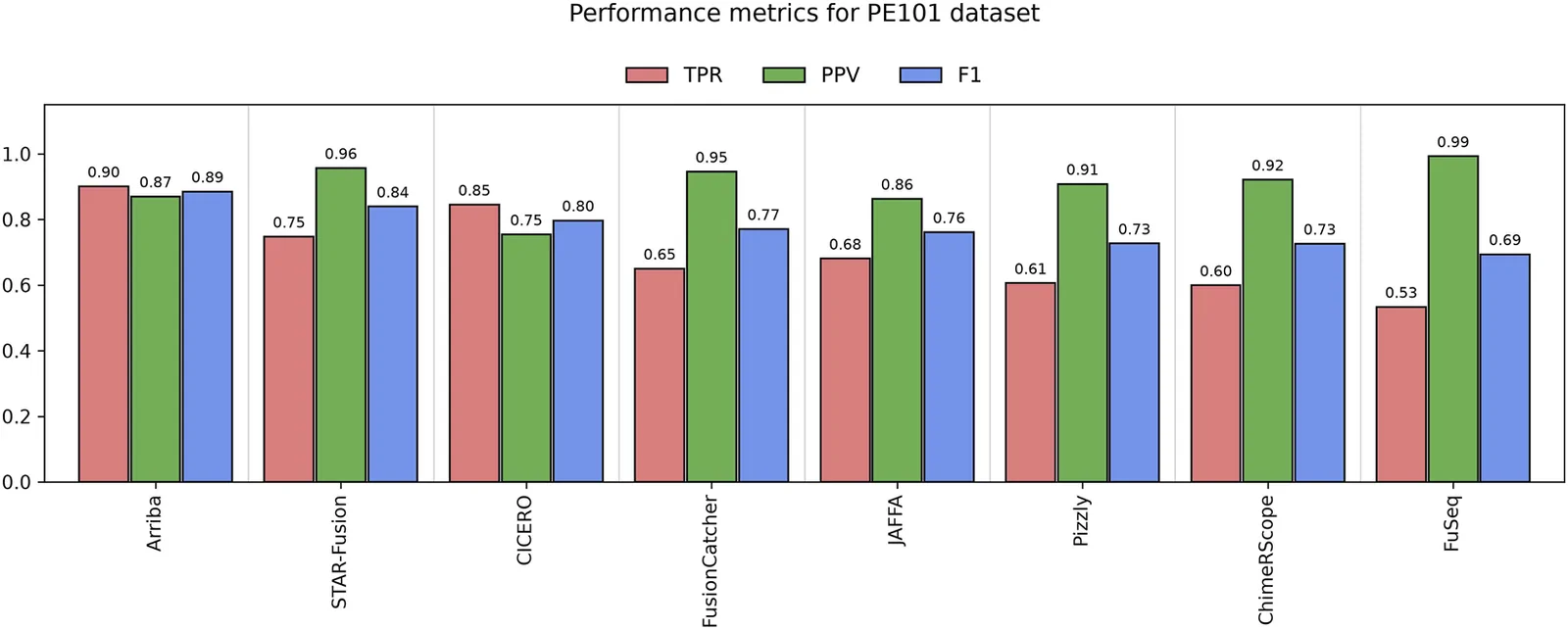
TPR, PPV, and F1-score for the simulated PE101 dataset, which contains 500 transcript fusions across 5 samples.

**FIGURE 6:**
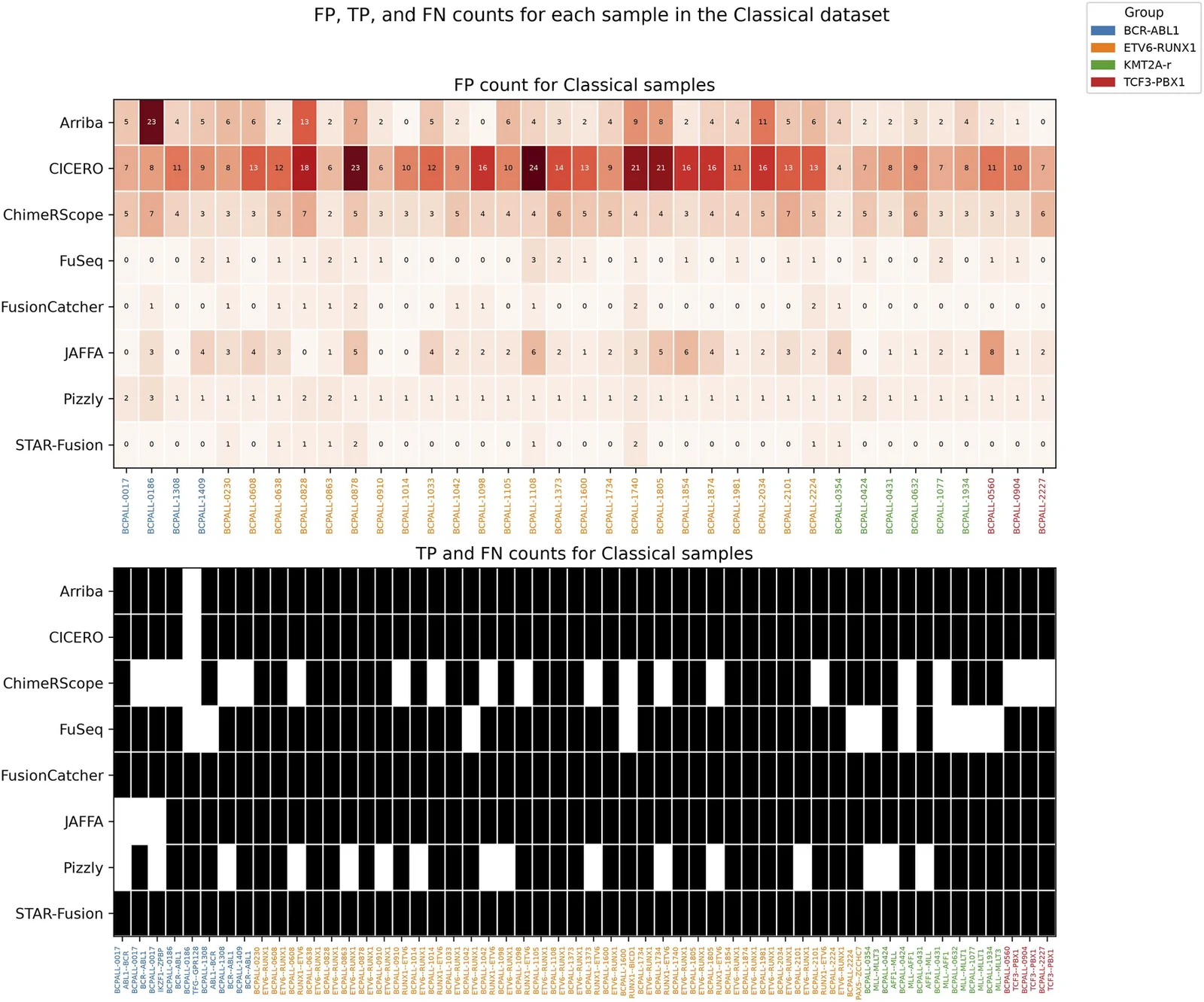
FP, TP, and FN counts for each sample in the Classical dataset. The numbers labeling each sample in the x-axis refer to their identifiers as presented in the supplementary table containing the transcript fusions they harbor as well as the validation methods.

**FIGURE 7:**
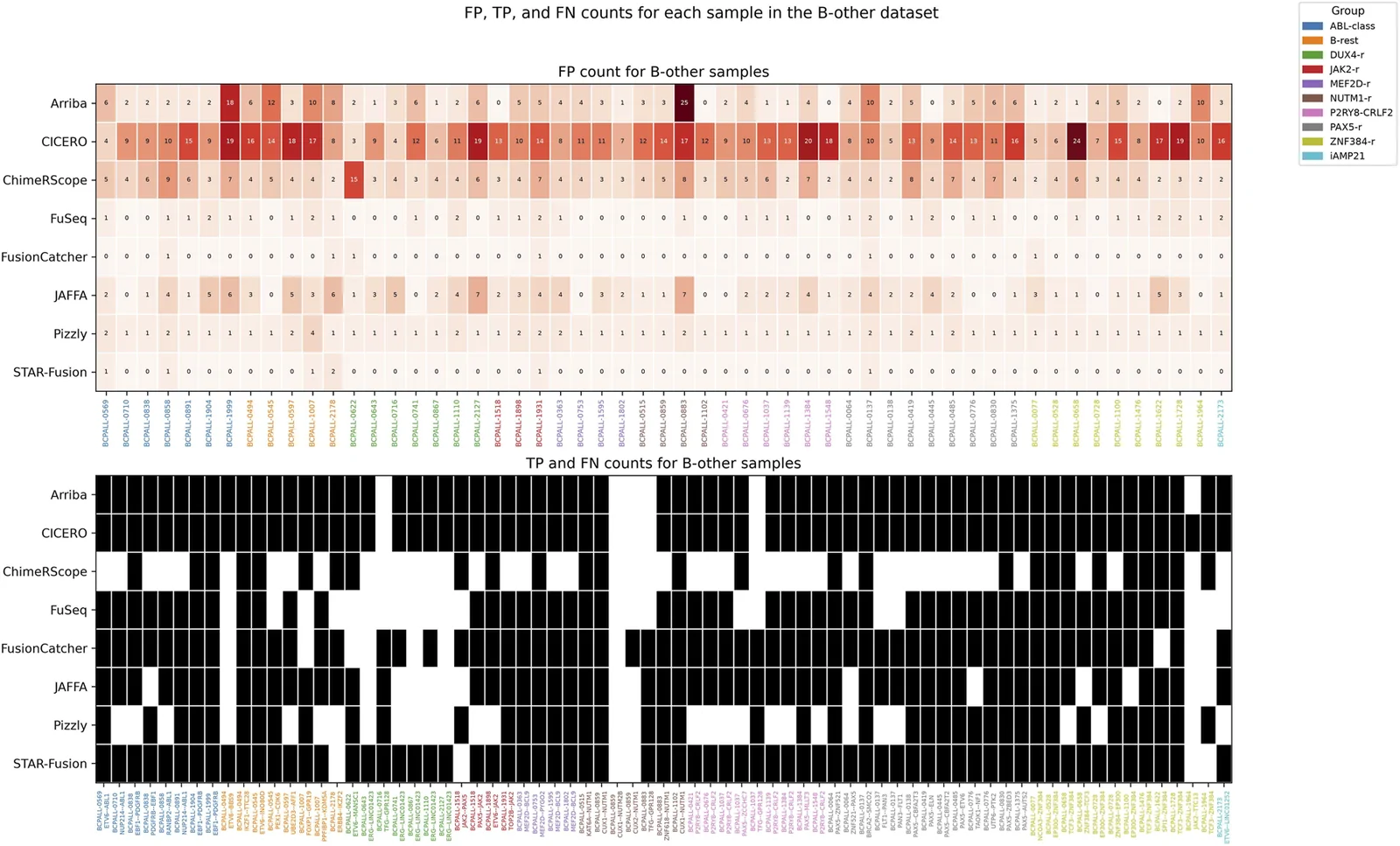
FP, TP, and FN counts for each sample in the B-other dataset. The numbers labeling each sample in the x-axis refer to their identifiers as presented in the supplementary table containing the transcript fusions they harbor as well as the validation methods.

### 3.1 Performance on simulated datasets

Regarding the areas under the precision-recall curves (AUC) shown in Figure 2, we observe a clear separation in mean value among the tools: Arriba, STAR-Fusion, CICERO, FusionCatcher, JAFFA, ChimeRScope, Pizzly, and FuSeq, ranked in strictly descending order for the PE101 dataset, while the order was Arriba, STAR-Fusion, FusionCatcher, Pizzly, ChimeRScope, JAFFA, FuSeq, and CICERO for the PE50 dataset. Every pairwise difference between tools was statistically significant, with a q -value less that 0.05 under the Benjamini–Hochberg corrected Mann-Whitney U test, with the sole exception of JAFFA and ChimeRScope on the PE101 dataset, which did not differ significantly (q=0.053) , indicating markedly separable rankings, with just one tie.

Overall, the alignment-based tools Arriba and STAR-Fusion had superior F1-score on both datasets (see Figures 4, 5): Arriba scored 84% on PE50 and 89% on PE101, and STAR-Fusion scored 80% on PE50 and 84% on PE101. The remaining tools stood in the interval [60%,80%) . We note that the TPR of every tool improved with read length, while PPV remained stable (with negligible losses) and above 85% (included), which is reflected in the mean AUC of the PR curves of the tools (see Figure 2). This data is summarized in Table 2. The only exception is Pizzly, which seems to be immune to read length. This indicates that every tool retrieved more true fusions with increased read length, which is an expected outcome since longer reads carry more information.

**TABLE 2:** Improvement of fusion detection methods with increased read length on the PE101 dataset compared to the PE50 dataset. Mean AUC of PR curves was computed over 100 random samplings, each selecting 50% of the samples in the dataset uniformly at random and computing the AUC of the PR curve in this subset.

Method	F1-score	TPR	PPV	Mean AUC
CICERO	+18%	+36%	−11%	+29%
JAFFA	+11%	+17%	−3%	+11%
FuSeq	+7%	+8%	+1%	+8%
Arriba	+5%	+8%	+2%	+8%
STAR-fusion	+4%	+6%	+0%	+6%
FusionCatcher	+2%	+3%	−1%	+3%
ChimeRScope	+1%	+2%	−1%	+3%
Pizzly	+0%	−1%	+1%	−1%

Notably, CICERO and JAFFA had the greatest discrepancy in F1-score and TPR between the PE50 and PE101 datasets. Indeed, the strategy employed by JAFFA is different depending on read length (as explained in Section 2.1): for the PE50 dataset, it first assembles the raw reads into transcripts before mapping against the transcriptome, while for the PE101 dataset, it maps the raw reads directly. CICERO, on the other hand, assembles a subset of raw reads regardless of read length. The inferior accuracy of both methods on the PE50 dataset is likely due to the degradation of the assembly routine on shorter reads. This behavior has been reported by Haas et al. (2019), in which assembly-based tools were also assessed.

### 3.2 Performance on real targeted RNA-seq dataset

In the targeted RNA-seq dataset, we again note a clear difference in mean AUC among the tools (see Figure 2): STAR-Fusion, FusionCatcher, FuSeq, Pizzly, JAFFA, Arriba, ChimeRScope, and CICERO ranked in strictly descending order of mean AUC, and every pairwise difference between tools was statistically significant ( q<0.05 , Benjamini–Hochberg corrected Mann-Whitney U tests), indicating a fully separable ranking with no ties.

Moreover, examining each tool’s ranking across the two settings reveals a more precise picture of robustness than the raw AUC gap alone. STAR-Fusion was the only tool to rank among the top two performers in both settings—first among the eight tools on the targeted RNA-seq dataset (89.9% mean AUC) and second on the simulated dataset (69.1%), behind only Arriba. By contrast, Arriba (first on simulated, sixth on targeted) and FusionCatcher (second on targeted, third on simulated) each occupied a top-tier position in only one setting. We note that FuSeq showed the smallest absolute gap in mean AUC between the two settings (14.2 percentage points), but this reflects consistently mediocre-to-low performance in both (63.5% and 49.3%, the latter the lowest of all eight tools on the simulated dataset) rather than robust high performance; a small gap alone is therefore not sufficient evidence of a tool’s practical utility.

As for F1-scores (see Figure 3), STAR-Fusion (91%), FusionCatcher (88%) scored the highest, followed by FuSeq (66%). The remaining tools, namely, Pizzly (50%), JAFFA (48%), Arriba (37%), ChimeRScope (20%), and CICERO (18%), spanned a broad intermediate-to-low range.

Regarding PPV, the tools scored across all samples as follows: STAR-Fusion scored 87%; FusionCatcher scored 85% ; FuSeq scored 59% ; Pizzly scored 42% ; JAFFA scored 33% ; and CICERO, ChimeRScope, and Arriba scored below 30% (10%, 13%, and 23%, respectively).

Regarding TPR, the tools scored across all samples as follows: CICERO and STAR-Fusion stood at 95%; Arriba stood at 94%; FusionCatcher stood at 92%; JAFFA stood at 85%; FuSeq stood at 74%; Pizzly stood at 64%; and ChimeRScope stood at 47%.

The overall TP and FN counts for the Classical and B-other samples differ, as can be seen in Figures 6, 7, respectively. In the Classical samples, FusionCatcher and STAR-Fusion were the only tools that retrieved every true fusion. Arriba and CICERO missed just the secondary *TFG::GPR128* event in the same BCR-ABL1 sample for both tools, while JAFFA missed three fusions, all concentrated in a single BCR-ABL1 sample (BCPALL-0017) rather than spread across the KMT2A-r group. Moreover, the alignment-free tools—Pizzly, ChimeRScope, and FuSeq—were the least capable of retrieving every true fusion across the Classical samples and, hence, yielded the highest number of FNs.

As for the B-other samples, we expected a loss in accuracy across all tools because these samples harbor a broader diversity of transcript fusions (as described in Section 2.3).

In particular, most tools missed the transcript fusions in the DUX4-r group that involve the *ERG* gene. These events are associated with complex genomic rearrangements involving DUX4 and secondary alterations affecting ERG, which may not produce canonical fusion transcript signatures detectable by standard tools.

Indeed, these fusions are due to losses in the sequence of this gene, and hence they lack the sequence pattern sought by most fusion detection tools. Here, we note that STAR-Fusion was the only tool to retrieve every DUX4-r fusion; Arriba and CICERO each missed just the secondary *TFG::GPR128* fusion, the same non-canonical, population-polymorphic event also present in the BCR-ABL1 Classical sample above, suggesting this specific fusion is difficult for these two alignment-based tools regardless of clinical context. Overall, we also note that the alingment-based tools—FusionCatcher, JAFFA, Arriba, STAR-Fusion, and CICERO—retrieved the highest number of TPs, while the alignment-free tools yielded the highest number of FNs.

### 3.3 Impact of truth-set validation status on tool performance

As noted in Section 2.3, 29 of the 62 fusion transcripts in the targeted RNA-seq truth set were independently confirmed by an orthogonal method, while the remainder were included on the basis of detection by at least three computational methods. To assess whether this distinction affects our conclusions, we repeated the benchmark restricting the ground truth to only the 29 orthogonally validated fusions, and compared the results against the full 62-fusion set.

Mean AUC and F1-score dropped for every tool under the validated-only truth set (Table 3). The decline was most pronounced for the best-performing tools: STAR-Fusion’s F1-score fell from 0.91 to 0.71, and its mean AUC from 0.90 to 0.63. FusionCatcher showed a similar pattern, with F1-score decreasing from 0.88 to 0.66 and mean AUC from 0.85 to 0.53. The relative ranking of tools was largely preserved between the two settings: STAR-Fusion, FusionCatcher, and FuSeq remained the top performers by AUC in both cases, while CICERO remained the weakest overall; ChimeRScope, Arriba, and CICERO occupied the bottom of the ranking in both settings, though the ordering among the first two was not stable given their closely overlapping AUC distributions under the validated-only truth set. This indicates that a portion of the tools’ apparent performance in the full-set benchmark is attributable to fusions whose ground-truth status rests on computational consensus rather than independent confirmation, and that this portion of the truth set may inflate the estimated accuracy of tools that share similar detection strategies with the majority-vote process by which those fusions were originally selected.

**TABLE 3:** Mean PR-AUC and F1-score for each tool, computed against the full 62-fusion truth set and the 29-fusion orthogonally-validated subset.

Tool	AUC (full)	AUC (validated)	F1 (full)	F1 (validated)
STAR-fusion	0.90	0.63	0.91	0.71
FusionCatcher	0.85	0.53	0.88	0.66
FuSeq	0.64	0.50	0.66	0.51
Pizzly	0.30	0.22	0.50	0.38
JAFFA	0.29	0.21	0.48	0.34
Arriba	0.20	0.15	0.37	0.26
ChimeRScope	0.16	0.15	0.20	0.16
CICERO	0.08	0.06	0.18	0.12

### 3.4 Computational resource usage

Runtime and peak memory usage were measured for each tool across all 92 targeted RNA-seq samples (Table 4; full per-sample data available as Supplementary Material). Pizzly and Arriba were the fastest and least memory-intensive tools, completing in under 2 minutes with peak memory below 5 GB, consistent with their reliance on pseudoalignment and an efficient genome-alignment pipeline, respectively. STAR-Fusion required the most memory of any tool (median 37.9 GB) but showed the narrowest memory range across samples, reflecting the fixed cost of loading its reference genome library rather than sample-dependent variation. CICERO and JAFFA were the slowest tools by a wide margin (median 25 and 24 min, respectively), with the widest runtime ranges observed (up to 67 and 81 min for the most demanding samples), likely reflecting their local-assembly-based detection strategies, whose computational cost scales with the complexity of a given sample’s reads. ChimeRScope showed the widest memory range of any tool (19.6–41.8 GB), approaching but not exceeding its configured 40 GB heap limit.

**TABLE 4:** Computational resource usage across the targeted RNA-seq dataset ( n=92 samples).

Tool	Median time	Time range	Median memory	Memory range
Pizzly	0m56s	0m40s–2m7s	850 MB	849–866 MB
Arriba	1m12s	0m59s–3m10s	4,343 MB	4,292–4,640 MB
FuSeq	1m57s	1m29s–5m10s	7,686 MB	7,656–7,724 MB
Star-fusion	4m15s	3m10s–10m2s	37,860 MB	37,520–37,910 MB
FusionCatcher	4m20s	3m5s–44m7s	6,570 MB	3,468–15,600 MB
ChimeRScope	6m9s	5m47s–11m46s	29,815 MB	19,570–41,820 MB
JAFFA	23m37s	2m6s–80m57s	13,890 MB	4,727–17,280 MB
CICERO	24m58s	5m1s–67m26s	7,254 MB	6,935–7,346 MB

### 3.5 Breakpoint-level agreement between tools

Among the 111 targeted-dataset fusion instances detected as true positives by two or more of the six coordinate-reporting tools, the large majority showed strong agreement: 44 (39.6%) showed unanimous agreement within 5bp, and a further 59 (53.2%) showed a clear majority position with one or more tools deviating from it, for a combined 92.8% showing consensus agreement. Only 8 instances (7.2%) showed a genuine tie or three-way split with no clear consensus among three or more tools, most often reflecting real, nearby but biologically distinct exon-junction choices (e.g., the ETV6:RUNX1 fusion, where three tools converged on one splice junction and two on another, roughly 6 kb apart). Notably, the six tools were not equally likely to be the outlier when one existed. Arriba deviated from the majority position in 51 of 111 comparisons in which it participated (45.9%), and CICERO in 37 of 110 (33.6%), compared to 15.8%, 15.2%, and 9.7% for JAFFA, FuSeq, and FusionCatcher, respectively, and just 0.9% (1 of 108) for STAR-Fusion. This large disparity between two alignment-based, genome-reference tools (Arriba and STAR-Fusion) suggests the difference is not attributable to alignment strategy alone, but to specific implementation choices in how each tool determines or reports the precise breakpoint position.

One apparent disagreement merits specific mention. For the P2RY8:CRLF2 fusion, a well-characterized driver in B-other ALL, five tools reported the breakpoint on chromosome X, while JAFFA reported the homologous position on chromosome Y. Both genes lie within the pseudoautosomal region (PAR1), which is genuinely duplicated in sequence between the X and Y chromosomes; JAFFA’s call is therefore not an error, but reflects a real ambiguity in the reference genome for this specific genomic region.

The complete per-fusion breakpoint comparison, including each tool’s exact reported coordinates, is available as Supplementary Material.

## 4 Discussion

Haas et al. (2019) presented benchmarks on four breast cancer cell lines: BT474, KPL4, MCF7, and SKBR3 (Maher et al., 2009; Edgren et al., 2011; Asmann et al., 2011; Kangaspeska et al., 2012). Singh and Li (2021) also presented a benchmark on these datasets, in addition to other ones. We did not assess the tools on bulk RNA-seq samples such as these, so our results for the targeted RNA-seq samples complement current benchmarks on real data. The only tool that we assess here that has not been assessed in these studies is CICERO. Such studies often report the superior performance of Arriba and STAR-Fusion over most tools on bulk RNA-seq datasets (Haas et al., 2019; Singh and Li, 2021). Our results on the targeted RNA-seq dataset, however, point out that STAR-Fusion, FusionCatcher, and FuSeq had superior performance when considering TPR, PPV, and F1-score altogether. Moreover, we noted that the PR curves of STAR-Fusion had high mean AUC on the targeted RNA-seq and simulated datasets, which indicates that this tool is more robust to handle different types of short-read assays. We did not observe this kind of performance in the remaining tools assessed here.

Fusion detection performance is influenced not only by algorithmic design but also by the structural characteristics of the underlying rearrangements. In particular, events involving complex genomic regions such as DUX4 and the immunoglobulin heavy chain (IGH) locus remain challenging for short-read RNA-seq, due to repetitive sequences, variable breakpoints, and non-templated insertions that impair the alignment of chimeric reads (Thomson et al., 2025). In addition, incomplete annotation and stringent filtering criteria may further reduce sensitivity, especially for non-canonical signals such as those observed in DUX4-rearranged cases involving ERG (Yasuda et al., 2016).

We notice that the datasets of real cancer samples used in standard bechmarks do not completely represent the broad diversity of transcript fusions associated with other types of cancer. Moreover, several studies have proposed fusion detection pipelines that employ a “majority vote” approach on top of multiple fusion detection tools, but they should be applied with caution to avoid missing important disease-related fusions detected by the minority (LaHaye et al., 2021; Vicente-Garcés et al., 2023; Apostolides et al., 2021). Instead of running the tools in a single round and applying a “majority vote” approach, an alternative strategy could be to split the analysis into two stages. First, fusion calling could be performed using tools that showed the highest precision in this benchmark (STAR-Fusion and FusionCatcher), which yielded far fewer false positives than the remaining six tools. Second, fusion calling could be performed using the remaining tools, retaining the fusions that intersect with the first stage as well as additional candidates supported by multiple tools in the second stage. These additional candidates may include non-canonical fusions that would otherwise be missed. For instance, CICERO’s local-assembly approach recovered the DUX4-r group’s ERG-related events despite its comparatively low overall precision. We note that the two best-precision tools identified here span both genome-reference (STAR-Fusion) and transcriptome-reference (FusionCatcher) architectures, indicating that reference type alone does not predict a tool’s false-positive rate in this setting. However, we emphasize that this strategy was not formally evaluated in this study and should be interpreted as a hypothesis motivated by the observed behavior of the tools rather than a validated recommendation. Further work would be required to assess its effectiveness.

We conclude discussing two limitations of our pipeline. First, adding new tools is a non-trivial task that requires programming knowledge, although done in a few steps. As for the current tools, we expect that they will remain compatible with the pipeline as long as their output format and command line interface does not change. Second, our ensemble approach for fusion detection is naive compared to other pipelines that perform a similar task (LaHaye et al., 2021; Vicente-Garcés et al., 2023; Apostolides et al., 2021): we do not apply a consensus algorithm, but rather keep all the retrieved fusions in a standardized format.

## 5 Conclusion

On the simulated datasets, our results agree with previous benchmarks: Arriba and STAR-Fusion had superior overall precision and recall (F1-scores of 0.86 and 0.82, respectively, and mean AUC of 0.75 and 0.69). On the targeted RNA-seq dataset, STAR-Fusion and FusionCatcher had superior overall performance by AUC (0.90 and 0.85, respectively), though both showed a marked decline in F1-score when scored against only the orthogonally validated subset of the truth set (Section 3.3). Considering recall alone on the targeted dataset, Arriba, STAR-Fusion, and CICERO retrieved the highest number of true fusions, although CICERO was accompanied by the highest number of false positives.

Of the tools benchmarked, only STAR-Fusion ranked among the top two performers in both the simulated and targeted RNA-seq settings (second and first, respectively), suggesting it is the most broadly robust choice across sequencing strategies. Arriba, despite ranking first on the simulated dataset, did not generalize to targeted data (AUC 0.20, sixth-ranked); FusionCatcher, despite ranking second on the targeted dataset, fell to third on the simulated dataset (AUC 0.60).

A limitation of this study is the absence of assembly-first methods, i.e., those that assemble all transcrips before mapping them against a reference. Since targeted RNA-seq datasets are much smaller than bulk RNA-seq ones, the computational resources demanded by assembly-first methods might be worth trying in this case. In addition, our analyses did not include bulk RNA-seq data of real cancer samples, which limits direct comparability with prior benchmarks. In conclusion, our results emphasize that tool performance is highly context-dependent and that evaluations on bulk RNA-seq data alone are insufficient to guide tool selection for targeted assays.

## Data Availability

The simulated datasets PE50 and PE101 for this study can be found, respectively, in https://zenodo.org/records/13354907 and https://zenodo.org/records/13359589. The code used for computing the mean AUC of PR curves is publicly available at https://github.com/fusiontranscripts/FusionBenchmarking/blob/devel/util/misc.

## Supplementary material

SUPPLEMENTARY DATA SHEET 1 Per-fusion breakpoint comparison.

SUPPLEMENTARY DATA SHEET 2 Runtime and peak memory per-sample, per-tool.

SUPPLEMENTARY DATA SHEET 3 List of samples, their fusions, and validation methods.
